# Supplementary material for: The association of sociodemographic characteristics and comorbidities with post-acute sequelae of SARS-CoV-2 in a Medicaid managed care population with and without HIV
Source: PLoS One. 2024 Jul 25;19(7):e0306322. doi: 10.1371/journal.pone.0306322 (PMC11271891; doi:10.1371/journal.pone.0306322)
Supplement: S1 File — (DOCX) [file pone.0306322.s001.docx]

**S1 File. All 49 PASC Symptoms by HIV Serostatus**

|  | **Total** | | **HIV Positive** | | **HIV Negative** | |  |
| --- | --- | --- | --- | --- | --- | --- | --- |
| **Symptom** | **N** | **%** | **N** | **%** | **N** | **%** | **Chi-Square** |
| **Weight loss** |  |  |  |  |  |  |  |
| No or less than five months | 132 | 95.7 | 102 | 95.3 | 30 | 96.8 |  |
| Six months or more | 6 | 4.3 | 5 | 4.7 | 1 | 3.2 | $x^{2}(2)=0.121$ |
| **Loss of appetite** |  |  |  |  |  |  |  |
| No or less than five months | 130 | 94.2 | 100 | 93.5 | 30 | 96.8 |  |
| Six months or more | 8 | 5.8 | 7 | 6.5 | 1 | 3.2 | $x^{2}(2)=0.484$ |
| **Fever, chills, sweats** |  |  |  |  |  |  |  |
| No or less than five months | 136 | 98.6 | 105 | 98.1 | 31 | 100.0 |  |
| Six months or more | 2 | 1.4 | 2 | 1.9 | 0 | 0 | $x^{2}(2)=0.588$ |
| **Hot flashes** |  |  |  |  |  |  |  |
| No or less than five months | 128 | 92.8 | 99 | 92.5 | 29 | 93.5 |  |
| Six months or more | 10 | 7.2 | 8 | 7.5 | 2 | 6.5 | $x^{2}(2)=0.038$ |
| **Fatigue** |  |  |  |  |  |  |  |
| No or less than five months | 111 | 80.4 | 87 | 81.3 | 24 | 77.4 |  |
| Six months or more | 27 | 19.6 | 20 | 18.7 | 7 | 22.6 | $x^{2}(2)=0.231$ |
| **Sleeping More** |  |  |  |  |  |  |  |
| No or less than five months | 122 | 88.4 | 95 | 88.8 | 27 | 87.1 |  |
| Six months or more | 16 | 11.6 | 12 | 11.2 | 4 | 12.9 | $x^{2}(2)=0.067$ |
| **Difficulty Sleeping** |  |  |  |  |  |  |  |
| No or less than five months | 117 | 84.8 | 88 | 82.2 | 29 | 93.5 |  |
| Six months or more | 21 | 15.2 | 19 | 17.8 | 2 | 6.5 | $x^{2}(2)=2.381$ |
| **Heat/cold intolerance** |  |  |  |  |  |  |  |
| No or less than five months | 122 | 88.4 | 94 | 87.9 | 28 | 90.3 |  |
| Six months or more | 16 | 11.6 | 13 | 12.1 | 3 | 9.7 | $x^{2}(2)=0.143$ |
| **Changing mood/impact on morale** |  |  |  |  |  |  |  |
| No or less than five months | 117 | 84.8 | 89 | 83.2 | 28 | 90.3 |  |
| Six months or more | 21 | 15.2 | 18 | 16.8 | 3 | 9.7 | $x^{2}(2)=0.951$ |
| **Body aches** |  |  |  |  |  |  |  |
| No or less than five months | 115 | 83.3 | 89 | 83.2 | 26 | 83.9 |  |
| Six months or more | 23 | 16.7 | 18 | 16.8 | 5 | 16.1 | $x^{2}(2)=0.008$ |
| **Rib cage pain** |  |  |  |  |  |  |  |
| No or less than five months | 128 | 92.8 | 98 | 91.6 | 30 | 96.8 |  |
| Six months or more | 10 | 7.2 | 9 | 8.4 | 1 | 3.2 | $x^{2}(2)=0.962$ |
| **Chest pressure** |  |  |  |  |  |  |  |
| No or less than five months | 124 | 89.9 | 98 | 91.6 | 26 | 83.9 |  |
| Six months or more | 14 | 10.1 | 9 | 8.4 | 5 | 16.1 | $x^{2}(2)=1.571$ |
| **Sharp sudden pain, chest burns** |  |  |  |  |  |  |  |
| No or less than five months | 129 | 93.5 | 100 | 93.5 | 29 | 93.5 |  |
| Six months or more | 9 | 6.5 | 7 | 6.5 | 2 | 6.5 | $x^{2}(2)=0.000$ |
| **Heart beating too fast, too slow, or irregular** |  |  |  |  |  |  |  |
| No or less than five months | 124 | 89.9 | 96 | 89.7 | 28 | 90.3 |  |
| Six months or more | 14 | 10.1 | 11 | 10.3 | 3 | 9.7 | $x^{2}(2)=0.010$ |
| **Dizziness** |  |  |  |  |  |  |  |
| No or less than five months | 121 | 87.7 | 93 | 86.9 | 28 | 90.3 |  |
| Six months or more | 17 | 12.3 | 14 | 13.1 | 3 | 9.7 | $x^{2}(2)=0.258$ |
| **Problem finding the right words** |  |  |  |  |  |  |  |
| No or less than five months | 117 | 84.8 | 91 | 85.0 | 26 | 83.9 |  |
| Six months or more | 21 | 15.2 | 16 | 15.0 | 5 | 16.1 | $x^{2}(2)=0.026$ |
| **Brain fog, difficulty concentrating** |  |  |  |  |  |  |  |
| No or less than five months | 109 | 79.0 | 84 | 78.5 | 25 | 80.6 |  |
| Six months or more | 29 | 21.0 | 23 | 21.5 | 6 | 19.4 | $x^{2}(2)=0.066$ |
| **Memory problems** |  |  |  |  |  |  |  |
| No or less than five months | 112 | 81.2 | 86 | 80.4 | 26 | 83.9 |  |
| Six months or more | 26 | 18.8 | 21 | 19.6 | 5 | 16.1 | $x^{2}(2)=0.192$ |
| **Pricking, tingling, or creeping feeling on the skin** |  |  |  |  |  |  |  |
| No or less than five months | 118 | 85.5 | 88 | 82.2 | 30 | 96.8 |  |
| Six months or more | 20 | 14.5 | 19 | 17.8 | 1 | 3.2 | $x^{2}(2)=4.096$* |
| **Impaired/decreased sense of touch** |  |  |  |  |  |  |  |
| No or less than five months | 130 | 94.2 | 102 | 95.3 | 28 | 90.3 |  |
| Six months or more | 8 | 5.8 | 5 | 4.7 | 3 | 9.7 | $x^{2}(2)=1.102$ |
| **Change/loss of taste** |  |  |  |  |  |  |  |
| No or less than five months | 125 | 90.6 | 99 | 92.5 | 26 | 83.9 |  |
| Six months or more | 13 | 9.4 | 8 | 7.5 | 5 | 16.1 | $x^{2}(2)=2.109$ |
| **Change/loss of smell** |  |  |  |  |  |  |  |
| No or less than five months | 132 | 95.7 | 104 | 97.2 | 28 | 90.3 |  |
| Six months or more | 6 | 4.3 | 3 | 2.8 | 3 | 9.7 | $x^{2}(2)=2.731$ |
| **Abdominal pain** |  |  |  |  |  |  |  |
| No or less than five months | 133 | 96.4 | 102 | 95.3 | 31 | 22.5 |  |
| Six months or more | 5 | 3.6 | 5 | 4.7 | 0 | 0.0 | $x^{2}(2)=1.503$ |
| **Nausea/vomiting** |  |  |  |  |  |  |  |
| No or less than five months | 131 | 94.9 | 102 | 95.3 | 29 | 93.5 |  |
| Six months or more | 7 | 5.1 | 5 | 4.7 | 2 | 6.5 | $x^{2}\left( 2 \right)=0.158$ |
| **Diarrhea** |  |  |  |  |  |  |  |
| No or less than five months | 129 | 93.5 | 100 | 93.5 | 29 | 93.5 |  |
| Six months or more | 9 | 6.5 | 7 | 6.5 | 2 | 6.5 | $x^{2}\left( 2 \right)=0.000$ |
| **Sore throat, tongue, mouth, trouble swallowing** |  |  |  |  |  |  |  |
| No or less than five months | 129 | 93.5 | 100 | 93.5 | 29 | 93.5 |  |
| Six months or more | 9 | 6.5 | 7 | 6.5 | 2 | 6.5 | $x^{2}\left( 2 \right)=0.000$ |
| **Ear pain** |  |  |  |  |  |  |  |
| No or less than five months | 132 | 95.7 | 102 | 95.3 | 30 | 96.8 |  |
| Six months or more | 6 | 4.3 | 5 | 4.7 | 1 | 3.2 | $x^{2}\left( 2 \right)=0.121$ |
| **Clogged ears** |  |  |  |  |  |  |  |
| No or less than five months | 132 | 95.7 | 101 | 94.4 | 31 | 100.0 |  |
| Six months or more | 6 | 4.3 | 6 | 5.6 | 0 | 0.0 | $x^{2}\left( 2 \right)=1.817$ |
| **Tinnitus** |  |  |  |  |  |  |  |
| No or less than five months | 121 | 87.7 | 93 | 86.9 | 28 | 90.3 |  |
| Six months or more | 17 | 12.3 | 14 | 13.1 | 3 | 9.7 | $x^{2}\left( 2 \right)=0.258$ |
| **Congested/runny nose** |  |  |  |  |  |  |  |
| No or less than five months | 126 | 91.3 | 98 | 91.6 | 28 | 90.3 |  |
| Six months or more | 12 | 8.7 | 9 | 8.4 | 3 | 9.7 | $x^{2}\left( 2 \right)=0.049$ |
| **Sensitivity to sound** |  |  |  |  |  |  |  |
| No or less than five months | 130 | 94.2 | 100 | 93.5 | 30 | 96.8 |  |
| Six months or more | 8 | 5.8 | 7 | 6.5 | 1 | 3.2 | $x^{2}\left( 2 \right)=0.484$ |
| **Dry eyes** |  |  |  |  |  |  |  |
| No or less than five months | 117 | 84.8 | 89 | 83.2 | 28 | 90.3 |  |
| Six months or more | 21 | 15.2 | 18 | 16.8 | 3 | 9.7 | $x^{2}\left( 2 \right)=0.951$ |
| **Blurry vision** |  |  |  |  |  |  |  |
| No or less than five months | 121 | 87.7 | 92 | 86.0 | 29 | 93.5 |  |
| Six months or more | 17 | 12.3 | 15 | 14.0 | 2 | 6.5 | $x^{2}\left( 2 \right)=1.274$ |
| **Sensitivity to light** |  |  |  |  |  |  |  |
| No or less than five months | 119 | 86.2 | 89 | 83.2 | 30 | 96.8 |  |
| Six months or more | 19 | 13.8 | 18 | 16.8 | 1 | 3.2 | $x^{2}\left( 2 \right)=3.$743 |
| **Bone or joint pain** |  |  |  |  |  |  |  |
| No or less than five months | 110 | 79.7 | 82 | 76.6 | 28 | 90.3 |  |
| Six months or more | 28 | 20.3 | 25 | 23.4 | 3 | 9.7 | $x^{2}(2)=2$.784 |
| **Heavy legs/swelling of the legs** |  |  |  |  |  |  |  |
| No or less than five months | 122 | 88.4 | 93 | 86.9 | 29 | 93.5 |  |
| Six months or more | 16 | 11.6 | 14 | 13.1 | 2 | 6.5 | $x^{2}\left( 2 \right)=1.032$ |
| **Muscle aches** |  |  |  |  |  |  |  |
| No or less than five months | 110 | 79.7 | 83 | 77.6 | 27 | 87.1 |  |
| Six months or more | 28 | 20.3 | 24 | 22.4 | 4 | 12.9 | $x^{2}(2)=1.349$ |
| **Neck, back, low back pain** |  |  |  |  |  |  |  |
| No or less than five months | 107 | 77.5 | 79 | 73.8 | 28 | 90.3 |  |
| Six months or more | 31 | 22.5 | 28 | 26.2 | 3 | 9.7 | $x^{2}(2)=3.753$ |
| **Bulging veins** |  |  |  |  |  |  |  |
| No or less than five months | 134 | 97.1 | 104 | 97.2 | 30 | 96.8 |  |
| Six months or more | 4 | 2.9 | 3 | 2.8 | 1 | 3.2 | $x^{2}(2)=0.015$ |
| **Unexplained bruising** |  |  |  |  |  |  |  |
| No or less than five months | 130 | 94.2 | 100 | 93.5 | 30 | 96.8 |  |
| Six months or more | 8 | 5.8 | 7 | 6.5 | 1 | 3.2 | $x^{2}(2)=0.484$ |
| **Swollen lymph nodes** |  |  |  |  |  |  |  |
| No or less than five months | 130 | 94.2 | 101 | 94.4 | 29 | 93.5 |  |
| Six months or more | 8 | 5.8 | 6 | 5.6 | 2 | 6.5 | $x^{2}(2)=0.031$ |
| **High or low blood pressure** |  |  |  |  |  |  |  |
| No or less than five months | 121 | 87.7 | 92 | 86.0 | 29 | 93.5 |  |
| Six months or more | 17 | 12.3 | 15 | 14.0 | 2 | 6.5 | $x^{2}(2)=1.274$ |
| **Dry/peeling skin** |  |  |  |  |  |  |  |
| No or less than five months | 128 | 92.8 | 97 | 90.7 | 31 | 100.0 |  |
| Six months or more | 10 | 7.2 | 10 | 9.3 | 0 | 0.0 | $x^{2}(2)=3.124$ |
| **Hair loss** |  |  |  |  |  |  |  |
| No or less than five months | 122 | 88.4 | 92 | 86.0 | 30 | 96.8 |  |
| Six months or more | 16 | 11.6 | 15 | 14.0 | 1 | 3.2 | $x^{2}(2)=2.732$ |
| **Skin rash** |  |  |  |  |  |  |  |
| No or less than five months | 130 | 94.2 | 99 | 92.5 | 31 | 100.0 |  |
| Six months or more | 8 | 5.8 | 8 | 7.5 | 0 | 0.0 | $x^{2}(2)=2.460$ |
| **Discoloration/swelling of hands or feet** |  |  |  |  |  |  |  |
| No or less than five months | 130 | 94.2 | 101 | 94.4 | 29 | 93.5 |  |
| Six months or more | 8 | 5.8 | 6 | 5.6 | 2 | 6.5 | $x^{2}(2)=0.031$ |
| **Changes in menstrual cycle** |  |  |  |  |  |  |  |
| No or less than five months | 135 | 97.8 | 105 | 98.1 | 30 | 96.8 |  |
| Six months or more | 3 | 2.2 | 2 | 1.9 | 1 | 3.2 | $x^{2}(2)=0.208$ |
| **Problems with urination** |  |  |  |  |  |  |  |
| No or less than five months | 131 | 94.9 | 101 | 94.4 | 30 | 96.8 |  |
| Six months or more | 7 | 5.1 | 6 | 5.6 | 1 | 3.2 | $x^{2}(2)=0.283$ |
| **Erectile dysfunction** |  |  |  |  |  |  |  |
| No or less than five months | 127 | 92.0 | 97 | 90.7 | 30 | 96.8 |  |
| Six months or more | 11 | 8.0 | 10 | 9.3 | 1 | 3.2 | $x^{2}(2)=1.227$ |
| Note. N = 138. HIV positive n = 107. HIV negative n = 31.  *p<.05, **p<.01, ***p<.001 | | | | | | | |
